# NDRG4 overexpression is associated with reduced apoptosis after intracerebral hemorrhage via the PI3K/Akt/GSK3β signaling pathway

**DOI:** 10.1038/s41598-025-33247-5

**Published:** 2026-01-03

**Authors:** Xiaoyan Wang, Zhimin Sun, Tianyu Dong, Pengfei Wang, Xiaoyang Zhang, Feng Mo, Liqiang Liu

**Affiliations:** 1 Department of Neurosurgery, The Second Hospital of Hebei Medical University, Shijiazhuang, 050000 Hebei China; 2 Department of Neurosurgery, Hebei General Hospital, Shijiazhuang, 050000 Hebei China; 3 Department of Neurosurgery, The Third Hospital of Shijiazhuang City, Shijiazhuang, 050000 Hebei China; 4 Department of Anatomy, Hebei Medical University, Shijiazhuang, 050000 Hebei China

**Keywords:** NDRG4, Apoptosis, PI3K/Akt signaling pathway, GSK3β, Brain injury, Neurology, Neuroscience

## Abstract

Intracerebral hemorrhage (ICH) is a severe form of stroke with high mortality, and apoptosis in the perihematomal region contributes to neurological deficits. This study aimed to investigate the role of NDRG4 in cerebral injury following ICH, focusing on apoptosis and related signaling pathways. A total of 242 male Sprague Dawley rats were used to establish a collagenase-induced ICH model and were allocated across four experiments to examine NDRG4 temporal expression, validate adenoviral overexpression, evaluate its effects on ICH outcomes, and probe PI3K/Akt/GSK3β signaling (6 rats per group). Neurological function, brain water content, TUNEL staining, Western blotting, and RT-qPCR were used to assess the effects of NDRG4 overexpression on ICH-induced brain injury and apoptosis. NDRG4 expression was significantly reduced in perihematomal brain tissue after intracerebral hemorrhage. In rats receiving adenoviral NDRG4 overexpression, neurological performance was significantly better than in ICH controls, and brain water content was significantly lower. NDRG4 overexpression was also associated with a significant reduction in TUNEL-positive cells, a significantly lower Bax/Bcl-2 ratio, and significantly decreased cleaved caspase-3 levels, while Bcl-2 levels were significantly higher. These biochemical and histological differences were accompanied by significantly increased phosphorylation of Akt (Ser473) and GSK3β (Ser9). Co-administration of wortmannin was associated with partial attenuation of these changes, suggesting that the observed effects may be related to activation of the PI3K/Akt/GSK3β signaling pathway. NDRG4 overexpression was associated with reduced perihematomal injury and improved neurological scores, partly associated with activation of the PI3K/Akt/GSK3β pathway. Further studies are warranted to delineate the specific cell types involved, the detailed mechanisms, and the translational relevance of these findings.

**Supplementary Information:**

The online version contains supplementary material available at 10.1038/s41598-025-33247-5.

## Introduction

Intracerebral hemorrhage (ICH) is one of the most devastating subtypes of stroke, associated with high mortality and long-term disability. It accounts for approximately 10–15% of all strokes in Western countries and 20–30% in Asian populations^1^. ICH has high morbidity and mortality that result from both primary and secondary injury mechanisms^2^. Patients often experience severe neurological dysfunction after surgery, which affects their quality of life. Over 70% of stroke survivors suffer from persistent locomotor impairments, and approximately 60% of these individuals subsequently develop delayed cognitive decline^3,4^. ICH caused by hematoma induces primary brain injury primarily through the mass effect of the intraparenchymal hematoma, which compresses and disrupts surrounding brain tissue, and through elevated intracranial pressure (ICP) resulting from hematoma expansion^5,6^. This is followed by secondary brain injury (SBI), triggered by the breakdown of red blood cells and other clot components. SBI involves complex molecular cascades, including inflammation, activation of apoptotic pathways, ischemia, disruption of the blood–brain barrier, and cerebral edema^7–9^. Among these processes, apoptosis is considered a predominant form of cell death in the perihematomal region^10,11^. Despite advances in stroke research, effective therapeutic options for ICH remain limited^12^. Given the lack of effective interventions, identifying neuroprotective targets that can modulate deleterious biochemical and molecular pathways—or enhance endogenous protective mechanisms—remains a critical focus in ICH research.

The serine/threonine kinase Akt is a key regulator of multiple physiological and pathological processes, including inflammation, cell survival, and apoptosis^13–15^. Once activated, Akt exerts anti-apoptotic effects by modulating several downstream targets, including inhibiting the glycogen synthase kinase-3β (GSK3β) activity^16,17^. When GSK3β is activated, it can exacerbate cellular damage and increase the activity of cleaved caspase-3 (CC3), an early marker of the execution phase of apoptosis^18^. Additionally, Akt’s anti-apoptotic mechanisms also involve the inhibition of specific proteins from the Bcl-2 family, highlighting the critical role of these factors in regulating cellular survival and death following acute brain injury^19^.

The N-Myc downstream-regulated gene (NDRG) family, consisting of NDRG1 to NDRG4, encodes a group of intracellular proteins that share significant amino acid sequence homology and exhibit distinct tissue-specific expression profiles^20–24^, has been implicated in a variety of physiological and pathological processes, including neurodevelopment, immune regulation, and tumor metastasis^25^. The NDRG family can be divided into two subfamilies based on sequence homology: NDRG1 and NDRG3 are in one subfamily, and NDRG2 and NDRG4 make up the second subfamily^26,27^. Unlike the ubiquitously expressed NDRG1–3, NDRG4 is predominantly expressed in the brain and heart, suggesting a specialized role in central nervous system function^21^. Accumulating evidence indicates that NDRG4 is involved in the regulation of cell proliferation, survival, apoptosis, and tumor invasion. In preclinical models, NDRG4-deficient mice exhibit impaired spatial learning, increased susceptibility to cerebral ischemia, growth retardation, and postnatal lethality^20,28,29^. In ischemia/reperfusion (I/R) rat models, NDRG4 expression is reduced in the brain, and adenovirus-mediated NDRG4 overexpression attenuates infarct size and mitigates I/R-induced neurological deficits by inhibiting neuronal apoptosis^30,31^. Mechanistically, NDRG4 also regulates brain-derived neurotrophic factor (BDNF) expression, which is essential for enhancing neuronal resistance to ischemia-induced injury^20^. In vitro studies in glioblastoma cell lines demonstrate that NDRG4 is essential for cell cycle progression and survival, and that NDRG4 knockdown induces G1 cell cycle arrest, apoptosis, and reduced invasion and migration^32^. Similarly, in two high-grade meningioma cell lines, NDRG4 is overexpressed in aggressive tumors, and its downregulation reduces cell proliferation, invasion, migration, and angiogenesis, indicating a critical role of NDRG4 in tumor progression^28^. Importantly, human studies support the clinical relevance of NDRG4 in neurological contexts. NDRG4 expression is decreased in brain tissues from patients with Alzheimer’s disease, suggesting a potential role in neurodegeneration^21^. In contrast, in glioblastoma patients, NDRG4 expression is significantly upregulated compared with normal brain tissue, and its expression correlates with tumor progression and patient survival^33^. Collectively, these findings indicate that NDRG4 functions are not restricted to preclinical models but also have translational relevance in human neurological diseases. Given that apoptosis in the perihematomal region is a major contributor to neurological deficits after intracerebral hemorrhage (ICH), the present study aimed to investigate the role of NDRG4 in cerebral injury following ICH, with a particular focus on its involvement in apoptosis and related signaling pathways.

## Materials and methods

### Animals and ICH model

A total of 242 healthy male Sprague Dawley (SD) rats (10–12 weeks old, 200–250 g) were used in this study, allocated across four independent experiments designed to examine NDRG4 temporal expression, validate adenoviral overexpression, assess ICH outcomes, and probe PI3K/Akt/GSK3β signaling (6 rats per group). Animals were housed under controlled environmental conditions (temperature 22 ± 2 °C, relative humidity 50–60%, and a 12 h light/dark cycle), with unrestricted access to food and tap water. The procedure for the rat ICH model was slightly modified from that described in previous publications^34^. Rats were anesthetized with an intraperitoneal injection of 1% pentobarbital sodium (40 mg/kg) and secured in a stereotactic frame (NeuroStar, Germany). Following a midline scalp incision, the bregma, lambda, and sagittal suture were exposed. A cranial hole (1 mm diameter) was drilled 0.24 mm posterior to the bregma and 3 mm lateral to the midline on the right side. Collagenase type IV (1 µL, 0.3 U/µL, Sigma-Aldrich, V900893) was infused into the right striatum via a PE tube at a rate of 0.2 µL/min using a microinjection pump (KDS310, KDS Co., USA). Sham-operated rats received an equal volume (1 µL) of normal saline without collagenase. Post-operative analgesia was provided with buprenorphine (0.05 mg/kg, s.c.) immediately after surgery and every 12 h for 48 h. Animals were monitored for pain, and additional analgesia was given if needed. Throughout the procedure, body temperature was maintained at 37 ± 0.5 °C using a heating blanket. For animals subjected to transcardial perfusion, deep anesthesia was induced by intraperitoneal injection of 1% pentobarbital sodium (≥ 50 mg/kg) until the loss of corneal and pedal reflexes was confirmed. Once a surgical level of anesthesia was achieved, rats were perfused transcardially with cold phosphate-buffered saline followed by 4% formaldehyde. After perfusion, brains were harvested and post-fixed for histological analyses. For animals that were not subjected to perfusion, euthanasia was performed by intraperitoneal injection of an overdose of pentobarbital sodium (≥ 150 mg/kg), in accordance with the recommendations of the American Veterinary Medical Association (AVMA) Guidelines for the Euthanasia of Animals (2020) and institutional ethical standards. All animal experiments were approved by the Animal Experimentation Ethics Committee of the Second Hospital of Hebei Medical University and conducted in accordance with the National Institutes of Health guidelines for the care and use of laboratory animals (2025-AE471). The study also adhered to the ARRIVE (Animal Research: Reporting of In Vivo Experiments) guidelines. All methods in this study were carried out in accordance with relevant guidelines and regulations.

#### Inclusion and exclusion criteria

All rats were eligible for inclusion if they met the following criteria: (1) body weight between 200 and 250 g at the time of surgery; (2) normal neurological function prior to experimentation; and (3) successful induction of intracerebral hemorrhage (ICH), confirmed by visible bleeding during surgery and subsequent hematoma formation. Animals were excluded based on pre-defined criteria: (1) failure of ICH induction (no detectable bleeding); (2) unexpected death unrelated to the experimental procedure before data collection; or (3) technical failure during surgery or sample processing that rendered the data unusable. In the present study, 8 rats were excluded due to failed ICH induction. All exclusions were documented and are summarized in Supplementary Materials 6 - Table S1.

### Experimental design

**Fig. 1:**
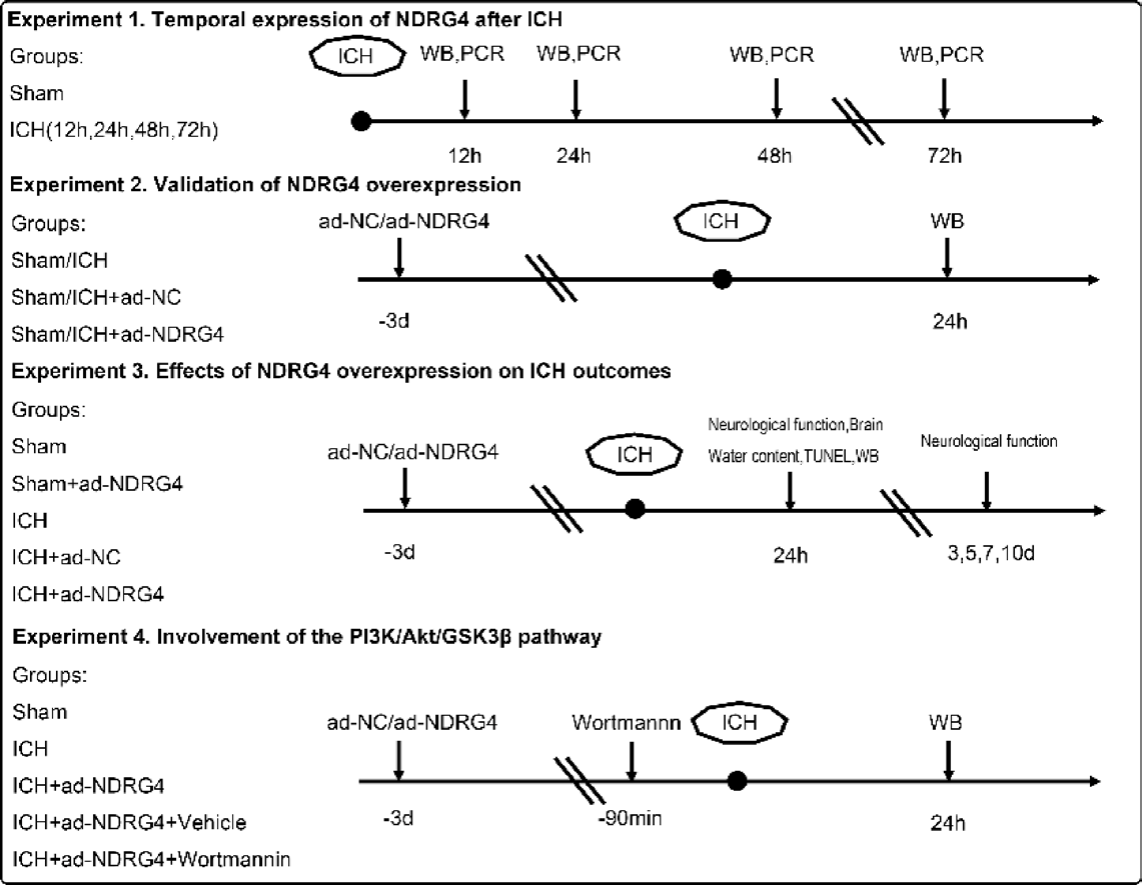
Experimental design and animal groups.

The study consisted of four independent experiments, each using a separate cohort of rats and designed for a specific purpose (Fig. 1).

#### Experiment 1: Temporal expression of NDRG4 after ICH

To examine the time course of NDRG4 expression, rats were divided into five groups (*n* = 6/group): Sham, ICH-12 h, ICH-24 h, ICH-48 h, and ICH-72 h. Brain tissues were collected at the indicated time points, and NDRG4 expression was analyzed using Western blot and PCR.

#### Experiment 2: Validation of NDRG4 overexpression

To confirm adenovirus-mediated NDRG4 overexpression, a separate cohort of rats was assigned to six groups (*n* = 6/group): Sham, Sham + ad-NC (adenoviral negative control vector), Sham + ad-NDRG4 (adenoviral vector encoding NDRG4), ICH, ICH + ad-NC, and ICH + ad-NDRG4. Three days prior to ICH induction, ad-NDRG4 or control vectors (1 × 10^8 U/mL, 0.1 mL) were stereotaxically injected into the right striatum with a 5-minute needle retention.

#### Experiment 3: Effects of NDRG4 overexpression on ICH outcomes

Another independent cohort of rats was divided into five groups: Sham, Sham + ad-NDRG4, ICH, ICH + ad-NC, and ICH + ad-NDRG4. Separate subsets of animals were used for different analyses. For each assay, 30 rats in total (six per group) were used—specifically, 30 for neurological assessments, 30 for brain water content measurement, 30 for TUNEL staining, and 30 for Western blot analysis. Neurological assessments were performed before ICH induction (pre) and on days 1, 3, 5, 7, and 10 after ICH, and these animals were not subjected to tissue collection. In contrast, brain tissues were harvested at 24 h post-ICH from additional animals for brain water content, TUNEL, and Western blot analyses.

#### Experiment 4: Involvement of the PI3K/Akt/GSK3β pathway

A fourth cohort of rats was assigned to five groups (*n* = 6/group): Sham, ICH, ICH + ad-NDRG4, ICH + ad-NDRG4 + Vehicle, and ICH + ad-NDRG4 + Wortmannin. Rats in the ICH + ad-NDRG4 + Wortmannin group received intravenous wortmannin (15 µg/kg; MedChemExpress, Shanghai, China) 90 min before ICH induction, while rats in the ICH + ad-NDRG4 + Vehicle group received an equal volume of sterile saline. Western blot analyses were performed 24 h after ICH.

### Neurobehavioral score

Neurological function was assessed within 10 days after ICH using the forelimb placement test, Bederson score, and Longa scale. In the forelimb placement test, when the vibrissae of the rats was brushed with the edge of the table corner, the normal rats could immediately put the ipsilateral forelimb above the table corner, whereas ICH rats were unable to perform this action due to motor function impairment of the haemorrhagic contralateral limb. Each rat was tested 10 times and the percentage of successful attempts was calculated^35^. The Bederson neurological score was determined through three sequential tests. In test 1, rats were suspended by the tail at a height of 10 cm to assess forelimb posture. Bilateral extension was scored as 0 and the assessment ended, whereas unilateral flexion was scored as 1, prompting further testing. In test 2, a lateral push was applied; reduced resistance was scored as 2 and led to test 3. In test 3, rats were observed during free movement, and persistent circling contralateral to the lesion was scored as 3^36^. The Longa score is evaluated on a 5-point scale:0 indicates no neurological impairment;1 signifies difficulty in stretching contralateral limbs post-hemorrhage;2 represents circling behavior;3 denotes tilting towards the hemiplegic side while awake; and 4 indicates an inability to walk and lack of consciousness^37^.

### Brain water content

As noted in a prior study, brain water content was measured using the dry-wet method^38^. The dissected rat brain was separated into the ipsilateral hemisphere, contralateral hemisphere, and cerebellum. Each part was first weighed to obtain the wet weight, then dried at 100 °C for 24 h to determine the dry weight. Brain water content was calculated as: (wet weight -dry weight)/wet weight× 100%.

### Western blot

Western blot was performed as previously described^34^. Striatal tissue surrounding the hematoma was collected and total protein quantified using a BCA kit (Report Biotech, China). Proteins underwent SDS-PAGE separation and were transferred onto PVDF membranes (Roche, Switzerland). After 5% milk blocking, membranes were incubated overnight at 4 °C with primary antibodies: NDRG4 (Cell Signaling, #9039), Bcl2 (Arigobio, ARG55188), Bax (Genetex, GTX109683), Caspase-3 (Proteintech, 19677-1-AP), Akt (ABways, CY5561), p-Akt (Ser473, ABways, CY6569), GSK3β (Affinity, AF5016), p-GSK3β (Ser9, Affinity, AF2016), α-Tubulin (Genetex, GTX628802), and GAPDH (ABways, AB0037). Membranes were then incubated with fluorescent secondary antibody (ROCKLAND, 611-145-002) for 2 h, imaged, and stored. Signal quantification was performed using ImageJ with α-Tubulin and GAPDH as references.

### RT-PCR

RT-PCR was performed as previously described^34^. Total RNA was extracted from brain tissues using the RNA Extraction Kit (Takara, Dalian, China) following the manufacturer’s protocol, cDNA was synthesized from RNA using the PrimeScript RT Kit (Takara), and subsequently used as a template for RT-qPCR, which was performed with the Premix Ex Taq II Kit (Takara) according to the manufacturer’s instructions. NDRG4 (Forward Primer: 5’- TCTTCCCTGATTTGGTGGAG-3’; Reverse Primer: 5’- CCAGAAGAGCTGAAGGTTGG-3’). With GAPDH as the internal reference, the relative expression of the gene was calculated by 2^−ΔΔCt method.

### Cryosections of rat brain

The brain samples used for TUNEL staining were prepared according to a previously described protocol^39^. At 24 h post-ICH, rats were deeply anesthetized with an intraperitoneal injection of 1% pentobarbital sodium (≥ 50 mg/kg) until the loss of corneal and pedal reflexes was confirmed. Once a surgical level of anesthesia was achieved, rats underwent transcardial perfusion with 200 mL of cold phosphate-buffered saline (PBS) followed by 500 mL of 4% formaldehyde. Brains were removed, postfixed in 4% formaldehyde at 4 °C for 24 h, then sequentially immersed in 20% and 30% sucrose solutions for cryoprotection. Tissues were frozen at -20 °C and cut into 5 μm coronal sections.

### Transferase-mediated Nick end labeling assay

To detect the apoptosis, TUNEL staining was performed at 24 h after ICH according to the manufacturer’s protocol (Beyotime, C1089). The stained sections were imaged with a fluorescent microscope (Zeiss, Axio Imager D2, American). As previously reported^40^, TUNEL-positive cells in the peri-hematoma region were quantified across six sections per brain at ×200 magnification using ImageJ software. The results were expressed as the percentage of TUNEL-positive cells.

### Statistical analysis

Data are presented as mean ± SD. Statistical analyses were performed using GraphPad Prism 9. Comparisons between two groups were conducted using Student’s t-test. Multiple-group comparisons of single-time-point measurements were performed using one-way ANOVA. Longitudinal behavioral assessments were analyzed using a mixed-model repeated-measures ANOVA. Sphericity was examined using Mauchly’s test, and when the assumption of sphericity was violated, Greenhouse–Geisser corrections were applied. Normality and homogeneity of variance were evaluated before performing parametric analyses. Given the small sample size (*n* = 6 per group), the limited power of normality tests was acknowledged, and distributional patterns were additionally inspected using Q–Q plots and histogram-based graphical methods in Prism. For key outcomes, non parametric Kruskal–Wallis analyses were also conducted to confirm result robustness. A P value < 0.05 was considered statistically significant.

## Results

### Mortality and exclusion

Of the 242 rats used in this study, 18 rats died during or after the procedures, resulting in an overall mortality rate of 7.44% (18/242). In addition, 8 rats were excluded because intracerebral hemorrhage induction failed (no detectable bleeding). A summary of group allocation, animal numbers, exclusions, and mortality rates is provided in Supplementary Materials 6 - Table S1.

### Endogenous NDRG4 expression was decreased following ICH

**Fig. 2:**
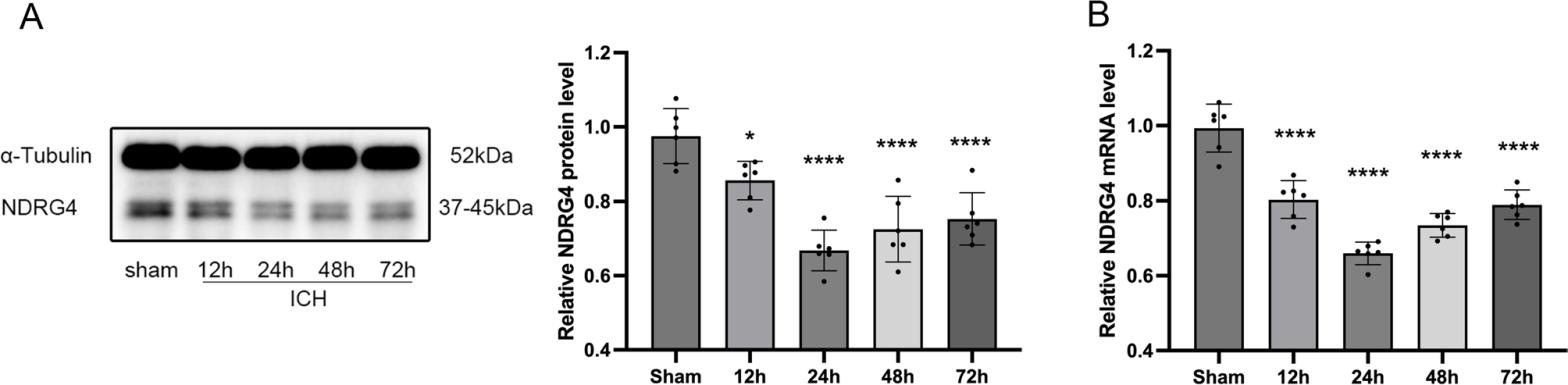
NDRG4 expression in the perihematomal region of ICH rat brains. (**A**) Western blot analysis of NDRG4 protein at 12, 24, 48, and 72 h after ICH (blots for NDRG4 and α-tubulin were obtained from the same gel under a single exposure; *n* = 6/group). (**B**) qRT-PCR analysis of NDRG4 mRNA (*n* = 6/group). One-way ANOVA with Tukey’s post-hoc test was used for statistical analysis. Data are presented as mean ± SD. *p < 0.05, ****p < 0.0001 vs. Sham.

To explore the temporal pattern of NDRG4 expression after ICH, we employed a previously established rat ICH model. At different time points after surgery, rats were sacrificed and perihematomal brain tissues were collected for analysis. Western blot analysis showed a significant reduction of NDRG4 protein levels after ICH, with the lowest expression observed at 24 h compared to the sham group. Data were assessed for normality (Shapiro–Wilk test, all *P* > 0.05) and variance homogeneity (Brown–Forsythe test, *P* = 0.7262; Bartlett test, *P* = 0.7699), with normality additionally evaluated using Q–Q plots; key results were verified using non-parametric Kruskal–Wallis tests, which yielded results consistent with ANOVA. One-way ANOVA revealed a significant overall effect (F(4,25) = 18.57, *P* < 0.0001), and post-hoc Tukey’s test indicated significant decreases compared to sham at 12 h (adjusted *P* = 0.0442), 24 h (adjusted *P* < 0.0001), 48 h (adjusted *P* < 0.0001), and 72 h (adjusted *P* < 0.0001) (Figs. 2A). Similarly, qRT-PCR analysis demonstrated a temporal decline in NDRG4 mRNA expression, with the lowest level at 24 h. One-way ANOVA confirmed a significant overall effect (F(4,25) = 45.95, *P* < 0.0001), and post-hoc Tukey’s multiple comparisons showed significant reductions compared to sham at 12 h (adjusted *P* < 0.0001), 24 h (adjusted *P* < 0.0001), 48 h (adjusted *P* < 0.0001), and 72 h (adjusted *P* < 0.0001) (Figs. 2B).

### NDRG4 overexpression and neurological outcomes after ICH

**Fig. 3:**
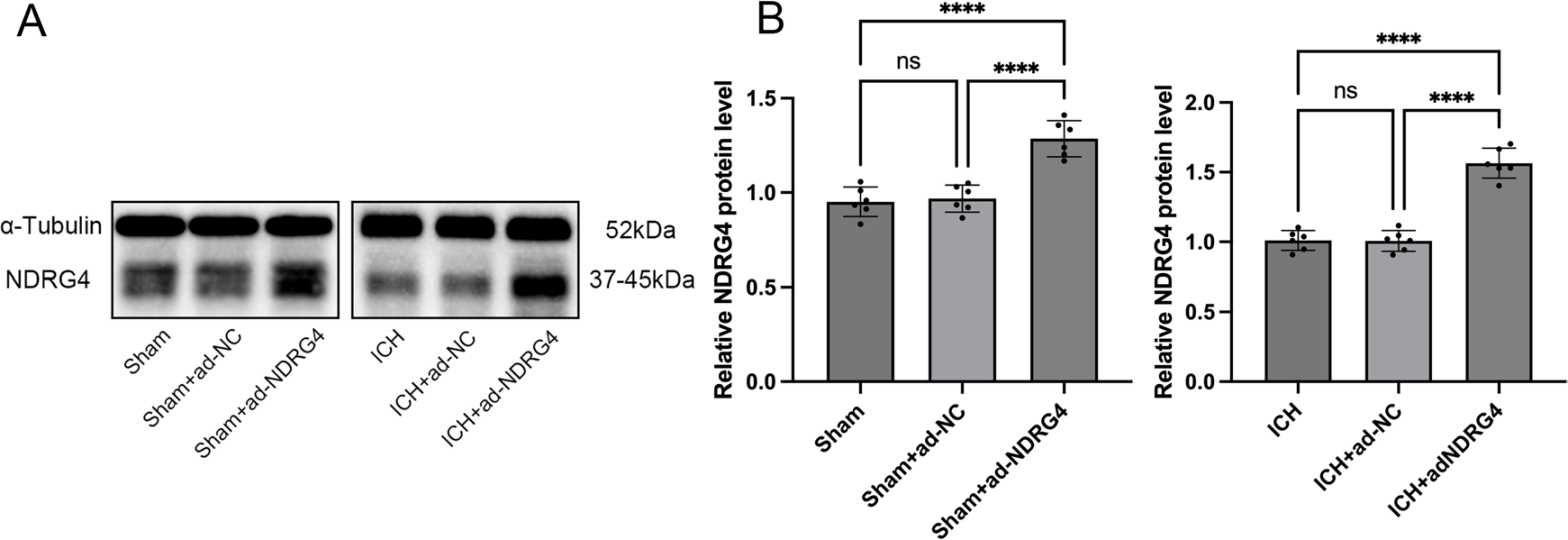
NDRG4 overexpression in sham and ICH rats. (**A**) Representative Western blots of NDRG4 and α-tubulin (same gel, single exposure). (**B**) Statistical analysis of NDRG4 detected via western blotting (*n* = 6/group). One-way ANOVA with Tukey’s post-hoc test was used for comparisons. Data are presented as mean ± SD. ****p < 0.0001.

**Fig. 4:**
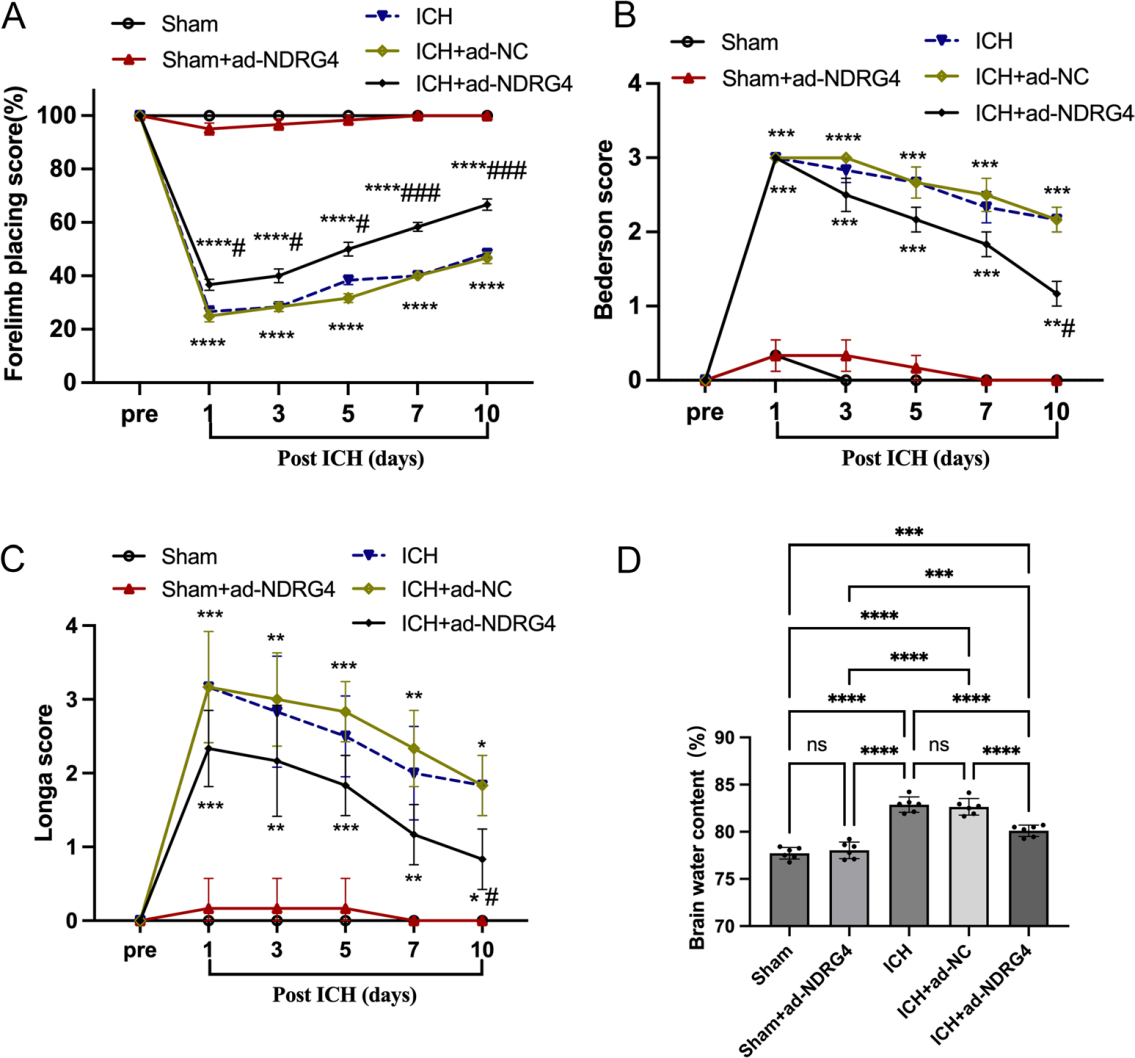
Neurological function was evaluated before ICH (pre) and at days 1, 3, 5, 7, and 10 after ICH using the Forelimb placing test (**A**), Bederson score (**B**), and Longa scale (**C**) (*n* = 6/group). Data were analyzed by mixed-model repeated-measures ANOVA with Tukey’s post-hoc test. (**D**) Brain water content (*n* = 6/group) was analyzed by one-way ANOVA with Tukey’s post-hoc test. Data are presented as mean ± SD. For panels A–C, * *p* < 0.05, ** *p* < 0.01, *** *p* < 0.001, **** *p* < 0.0001 vs. Sham; # *p* < 0.05, ### *p* < 0.001 vs. ICH. For panel D, significance between groups is indicated by *** *p* < 0.001, **** *p* < 0.0001.

To explore the potential role of NDRG4 in vivo, rats received adenovirus-mediated vectors encoding NDRG4 (ad-NDRG4) or control vectors (ad-NC). Western blot confirmed NDRG4 overexpression in both sham and ICH rats. Data met normality (Shapiro-Wilk test, all *P* > 0.05) and variance homogeneity (Brown-Forsythe and Bartlett tests, all *P* > 0.05), with normality additionally evaluated using Q–Q plots. In sham rats, one-way ANOVA showed a significant overall effect (F(2,15) = 31.29, *P* < 0.0001). Tukey’s post-hoc test indicated no difference between Sham and Sham + ad-NC (adjusted *P* = 0.9313), but Sham + ad-NDRG4 was significantly higher than both Sham (adjusted *P* < 0.0001) and Sham + ad-NC (adjusted *P* < 0.0001). In ICH rats, one-way ANOVA revealed a significant effect (F(2,15) = 82.97, *P* < 0.0001), with ICH + ad-NDRG4 elevated compared to ICH (adjusted *P* < 0.0001) and ICH + ad-NC (adjusted *P* < 0.0001), while ICH vs. ICH + ad-NC was not significant (adjusted *P* > 0.05). These results confirm effective NDRG4 overexpression in vivo (Fig. 3). Neurological function was evaluated before ICH (pre) and at days 1, 3, 5, 7, and 10 after ICH using the forelimb placement test, Bederson score, and Longa scale. Data were tested for normality (Shapiro–Wilk) and variance homogeneity (Brown–Forsythe). Statistical analysis was performed using a mixed-model repeated-measures ANOVA, with Time as the within-subject factor and Group as the between-subject factor, followed by Tukey’s post-hoc correction for multiple testing. In the forelimb placement test, ANOVA revealed significant main effects of Group (F(4,25) = 481.3, *P* < 0.0001) and Time (Geisser-Greenhouse corrected F(1.184, 34.32) = 44.61, *P* < 0.0001), as well as a Group × Time interaction. Post-hoc comparisons showed that ICH rats exhibited severe neurological deficits compared with sham at all post-injury time points (all *P* < 0.0001). Rats in the ICH + ad-NDRG4 group displayed significant functional improvement compared with ICH rats beginning on day 1 (all *P* < 0.05), with the effect persisting at days 3 and 5 (*P* < 0.05) and becoming more pronounced at days 7 and 10 (all *P* < 0.001). No significant differences were observed between ICH and ICH + ad-NC groups at any time point (Fig. 4A). Across the 10-day observation period, Bederson and Longa neurological scores exhibited significant main effects of time and group, as well as significant time group interactions (Bederson: time group interaction F(20,125) = 22.51, *p* < 0.0001; main effect of group F(4,25) = 183.0, *p* < 0.0001. Longa: time group interaction F(20,125) = 21.54, *p* < 0.0001; main effect of group F(4,25) = 65.69, *p* < 0.0001). Post hoc comparisons showed that NDRG4 overexpression did not improve neurological scores relative to ICH or ICH + ad-NC groups from day 1 to day 7 (all adjusted *p* > 0.05). However, by day 10, rats in the ICH + ad-NDRG4 group displayed significantly lower neurological deficit scores than those in the ICH and ICH + ad-NC groups for both Bederson and Longa assessments (all adjusted *p* < 0.05). These findings indicate that NDRG4 overexpression confers a delayed but measurable improvement in neurological recovery after ICH (Fig. 4B–C). In addition, brain water content, an indicator of cerebral edema, was elevated in the ICH group compared to the sham group. One-way ANOVA revealed a significant effect of treatment on brain water content (F(4,25) = 60.47, *P* < 0.0001), and Tukey’s multiple comparisons test with correction for multiple testing showed that ICH and ICH + ad-NC groups had significantly higher brain water content than Sham and Sham + ad-NDRG4 (adjusted *P* < 0.0001), whereas ICH + ad-NDRG4 rats exhibited reduced brain water content compared with ICH and ICH + ad-NC (adjusted *P* < 0.0001) but remained higher than Sham and Sham + ad-NDRG4 (adjusted *P* < 0.001); no significant differences were observed between Sham and Sham + ad-NDRG4 or between ICH and ICH + ad-NC (Fig. 4D). These results indicate that NDRG4 overexpression partially alleviates ICH-induced cerebral edema.

### NDRG4 overexpression is associated with reduced apoptosis after ICH

**Fig. 5:**
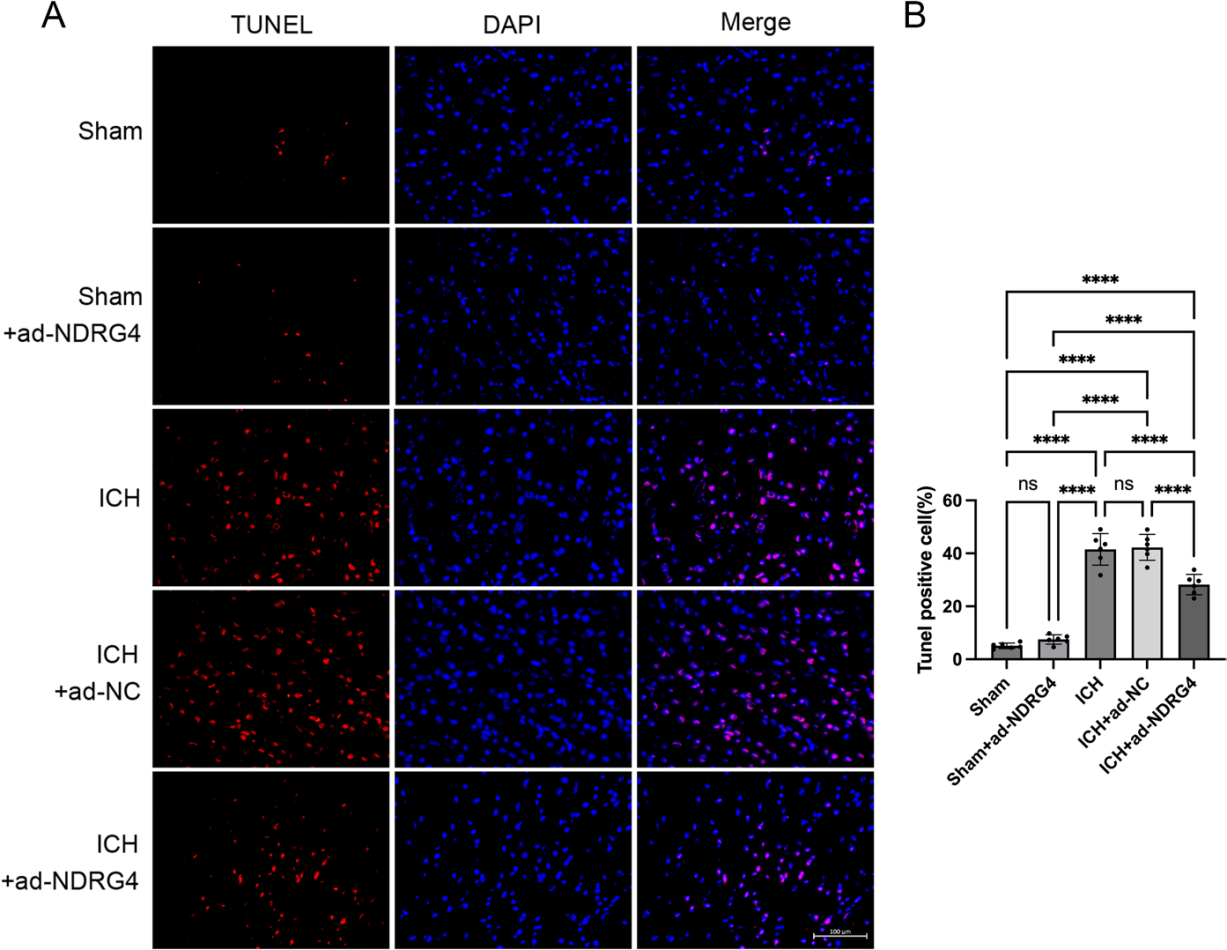
NDRG4 overexpression is associated with reduced apoptosis at 24 h after ICH. (**A**) TUNEL staining showing apoptotic cells in perihematomal brain regions. Scale bar = 100 μm. (**B**) Quantification of TUNEL-positive cells (*n* = 6/group). One-way ANOVA with Tukey’s post-hoc test was used to compare all groups. Data are presented as mean ± SD. ****p < 0.0001.

**Fig. 6:**
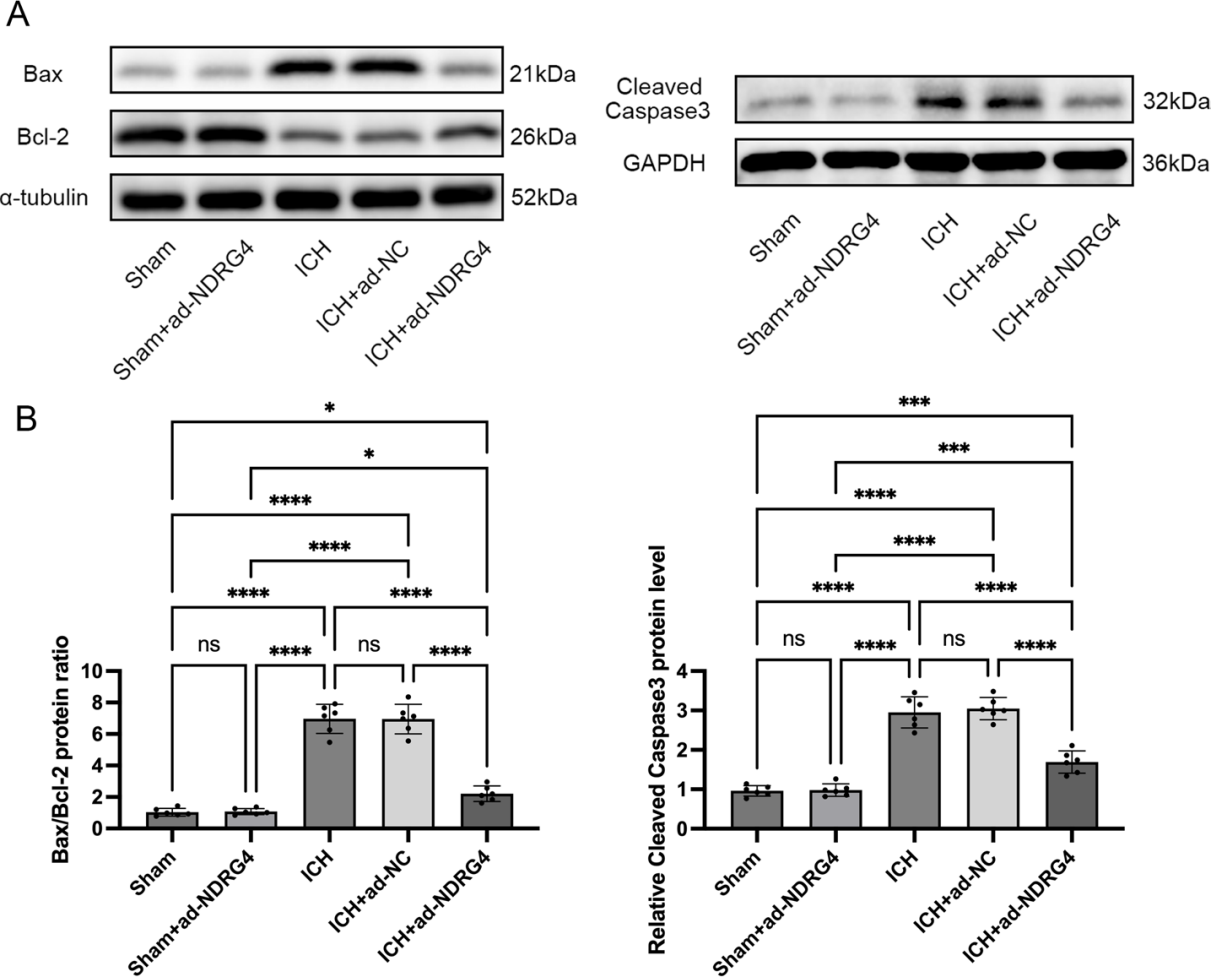
NDRG4 overexpression is associated with changes in apoptosis-related protein expression at 24 h after ICH. (**A**) Representative Western blots of Bax, Bcl-2, and cleaved caspase-3 (blots for Bax/α-tubulin, Bcl-2/α-tubulin, and cleaved caspase-3/GAPDH were obtained from the same gel under a single exposure). (**B**) Quantitative analysis of the Bax/Bcl-2 ratio and cleaved caspase-3 expression (*n* = 6/group). One-way ANOVA with Tukey’s post-hoc test was used for comparisons. Data are presented as mean ± SD. * *p* < 0.05, *** *p* < 0.001, ****P < 0.0001.

To examine the potential influence of NDRG4 on apoptosis. TUNEL staining was performed to evaluate apoptosis in perihematomal brain tissue 24 h after ICH. Data were normally distributed (Shapiro-Wilk test, all *P* > 0.05) and variances were homogeneous (Brown-Forsythe and Bartlett tests, all *P* > 0.05), with normality additionally evaluated using Q–Q plots. One-way ANOVA indicated significant differences among the five groups in TUNEL-positive cell counts (F(4,25) = 122.6, *P* < 0.0001). Tukey’s post-hoc test with correction for multiple comparisons showed that both the ICH and ICH + ad-NC groups exhibited markedly increased TUNEL-positive cells compared with the Sham and Sham + ad-NC groups (all *P* < 0.0001), indicating enhanced apoptosis. There was no significant difference between the ICH and ICH + ad-NC groups. NDRG4 overexpression (ICH + ad-NDRG4) significantly reduced TUNEL-positive cell counts relative to both ICH and ICH + ad-NC (all *P* < 0.0001), demonstrating its association with decreased apoptosis. As expected, the ICH + ad-NDRG4 group still displayed substantially more apoptotic cells than the Sham and Sham + ad-NDRG4 groups (all *P* < 0.0001), consistent with the injury model. Although the apoptosis proportion in Sham + ad-NDRG4 appeared slightly higher than Sham, this difference was not statistically significant (*p* = 0.8322; Fig. 5). To further investigate the underlying mechanisms, we assessed apoptosis-related proteins, including Bax, Bcl-2, and cleaved caspase-3. Data were normally distributed (Shapiro–Wilk test) and showed equal variance (Brown–Forsythe and Bartlett tests). One-way ANOVA revealed significant overall differences among groups in the Bax/Bcl-2 ratio (F(4,25) = 134.8, *P* < 0.0001) and cleaved caspase-3 expression (F(4,25) = 87.49, *P* < 0.0001). Post-hoc Tukey’s multiple comparisons analysis revealed distinct patterns of apoptotic signaling among the experimental groups. Compared with the sham group, both the ICH and ICH + ad-NC groups exhibited markedly increased Bax/Bcl-2 ratio (adjusted *P* < 0.0001 for both) and cleaved caspase-3 expression (adjusted *P* < 0.0001 for both), consistent with enhanced apoptosis after ICH (Fig. 6). In contrast, the sham + ad-NDRG4 group did not differ significantly from sham (Bax/Bcl-2: adjusted *P* > 0.05; cleaved caspase-3: adjusted *P* > 0.05), indicating that NDRG4 overexpression alone was not associated with changes in baseline apoptotic signaling. In the ICH + ad-NDRG4 group, Bax/Bcl-2 and cleaved caspase-3 levels were lower than those in the ICH and ICH + ad-NC groups (Bax/Bcl-2: adjusted *P* < 0.0001; cleaved caspase-3: adjusted *P* < 0.0001 for both comparisons), but remained higher than in sham (Bax/Bcl-2: adjusted *P* < 0.05; cleaved caspase-3: adjusted *P* < 0.001), suggesting that NDRG4 overexpression may be associated with a partial modulation of apoptotic signaling following ICH. Comparisons between ICH and ICH + ad-NC groups showed no significant differences (Bax/Bcl-2: adjusted *P* > 0.05; cleaved caspase-3: adjusted *P* > 0.05). These data indicate that apoptosis occurs in the perihematomal region after ICH, and that NDRG4 overexpression may contribute to protection, at least in part, through modulation of apoptotic signaling pathways.

### Possible involvement of the PI3K/Akt/GSK3β pathway in NDRG4-associated effects

**Fig. 7:**
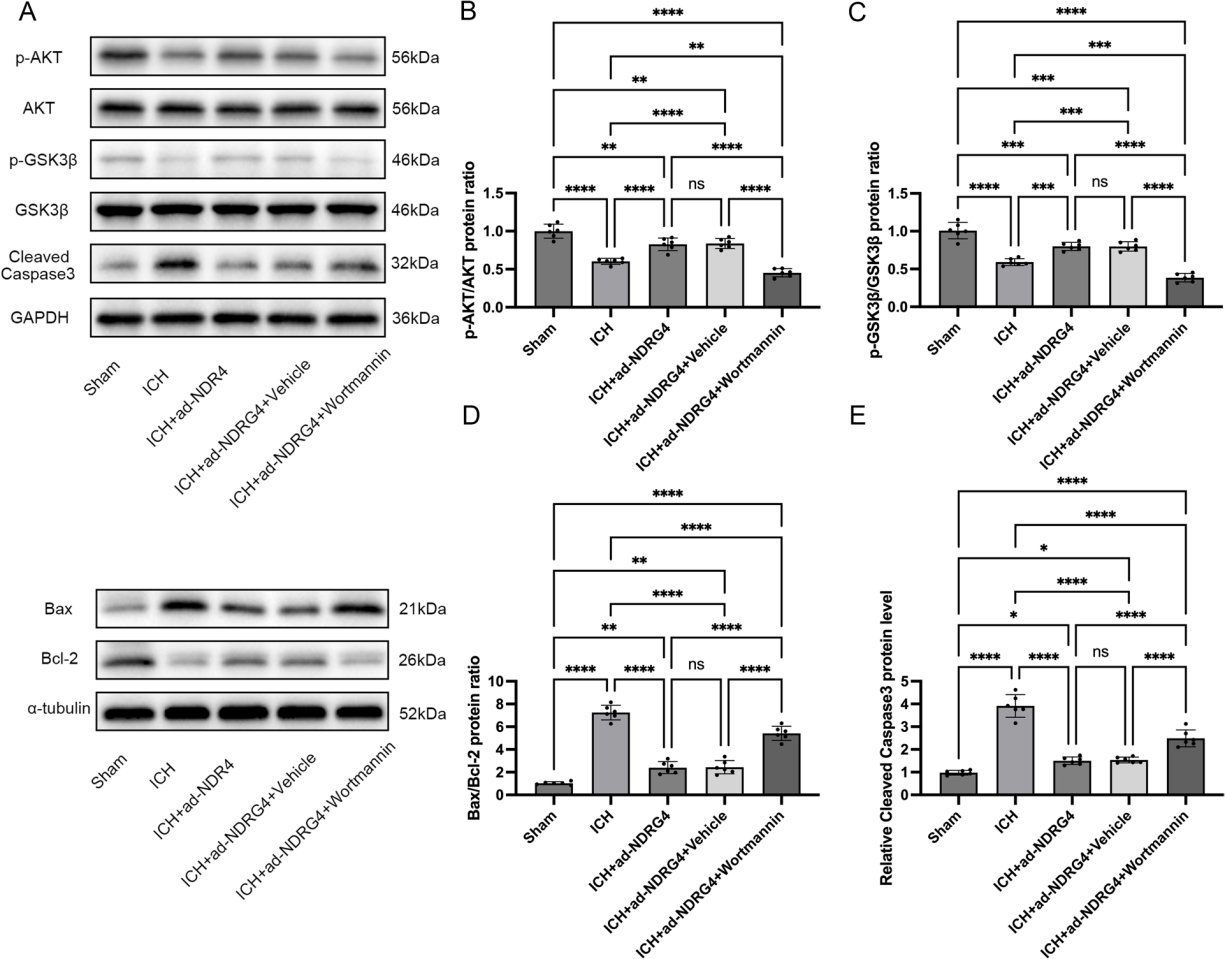
NDRG4 overexpression and its association with PI3K/Akt/GSK3β signaling and apoptosis. Representative Western blot images (**A**) and quantitative analyses of p-AKT/AKT (**B**), p-GSK3β/GSK3β (**C**), Bax/Bcl-2 (**D**), and cleaved caspase-3 (**E**) (*n* = 6/group). Blots for p-AKT/GAPDH, AKT/GAPDH, p-GSK3β/GAPDH, GSK3β/GAPDH, Bax/α-tubulin, Bcl-2/α-tubulin, and cleaved caspase-3/GAPDH were obtained from the same gel under a single exposure. Data are presented as mean ± SD. Statistical analysis was performed using one-way ANOVA followed by Tukey’s post hoc test. * *p* < 0.05, ** *p* < 0.01, ***p < 0.001.****p < 0.0001.

To explore whether the associations observed with NDRG4 overexpression might relate to the PI3K/Akt/GSK3β pathway, we measured p-Akt (Ser473), p-GSK3β (Ser9), Bax/Bcl-2 ratio, and cleaved caspase-3 levels by Western blotting. Data met normality (Shapiro-Wilk test, all *P* > 0.05) and variance homogeneity (Brown-Forsythe and Bartlett tests, all *P* > 0.05), with normality additionally evaluated using Q–Q plots. One-way ANOVA revealed significant differences in p-Akt (F(4,25) = 57.76, *P* < 0.0001) and p-GSK3β (F(4,25) = 71.0, *P* < 0.0001) levels among the groups. Post-hoc Tukey’s multiple comparisons analysis indicated that, compared with the sham group, the ICH group showed significantly lower p-Akt (adjusted *P* < 0.0001) and p-GSK3β (adjusted *P* < 0.0001) levels. The ICH + ad-NDRG4 and ICH + ad-NDRG4 + Vehicle groups exhibited intermediate levels of p-Akt and p-GSK3β, higher than those in the ICH group (p-Akt: adjusted *P* < 0.0001; p-GSK3β: adjusted *P* < 0.001) but lower than sham (p-Akt: adjusted *P* < 0.01; p-GSK3β: adjusted *P* < 0.001). In contrast, the ICH + ad-NDRG4 + Wortmannin group showed the lowest levels of p-Akt and p-GSK3β among all groups (p-Akt: adjusted *P* < 0.0001 versus sham and versus ICH + ad-NDRG4; p-GSK3β: adjusted *P* < 0.0001 for the same comparisons). No significant differences were observed between the ICH + ad-NDRG4 and ICH + ad-NDRG4 + Vehicle groups (adjusted *P* > 0.05 for both) (Figs. 7A–C). In parallel, ICH was associated with a pro-apoptotic profile, reflected by an increased Bax/Bcl-2 ratio and cleaved caspase-3 levels compared with the sham group (Bax/Bcl-2: F(4,25) = 135.6, *P* < 0.0001; cleaved caspase-3: F(4,25) = 91.78, *P* < 0.0001; post-hoc Tukey’s test). Bax/Bcl-2 ratio and cleaved caspase-3 levels in the ICH + ad-NDRG4 and ICH + ad-NDRG4 + Vehicle groups were significantly lower than those in the ICH group (Bax/Bcl-2: adjusted *P* < 0.0001; cleaved caspase-3: adjusted *P* < 0.0001) but remained elevated relative to sham (Bax/Bcl-2: adjusted *P* < 0.01; cleaved caspase-3: adjusted *P* < 0.05). Co-treatment with wortmannin was associated with increased Bax/Bcl-2 ratio and cleaved caspase-3 levels compared with ICH + ad-NDRG4 and ICH + ad-NDRG4 + Vehicle groups (Bax/Bcl-2: adjusted *P* < 0.0001; cleaved caspase-3: adjusted *P* < 0.0001). No significant differences were observed between the ICH + ad-NDRG4 and ICH + ad-NDRG4 + Vehicle groups (adjusted *P* > 0.05 for both) (Figs. 7A, D and E). Taken together, these findings indicate that the protective associations observed with NDRG4 overexpression may be associated with activation of the PI3K/Akt/GSK3β pathway.

## Discussion

This study provides new evidence that NDRG4 expression is significantly downregulated following ICH in rats, and that NDRG4 overexpression prior to ICH induction was associated with improved neurological scores, lower brain water content, and reduced markers of apoptosis. These associations appeared to be, at least in part, linked with activation of the PI3K/Akt/GSK3β pathway. While earlier studies have primarily focused on the role of NDRG4 in cerebral ischemia and brain tumors^20,21,32,41^, its involvement in hemorrhagic stroke has not been previously reported. Our results therefore extend the current understanding of NDRG4, highlighting its potential as a regulator of secondary brain injury after ICH.

Previous studies have shown that NDRG4 is involved in neuronal survival and apoptosis through regulation of pathways such as BDNF signaling and cell cycle control. It has been implicated in ischemic brain injury, glioblastoma, and Alzheimer’s disease, highlighting its diverse roles in neurological disorders. In this study, we provide novel evidence that experimental NDRG4 overexpression alleviates ICH-induced injury, thereby extending its neuroprotective relevance to hemorrhagic stroke.

In our rat ICH model, NDRG4 overexpression was associated with improved neurological performance across multiple behavioral tests and with lower brain water content, suggesting a potential reduction in cerebral edema. Brain edema is a critical component of secondary brain injury after ICH, contributing to increased intracranial pressure, disruption of cellular homeostasis, and exacerbation of tissue damage^10,42,43^. The reduction in brain edema observed in NDRG4-overexpressing animals may be partly attributable to its anti-apoptotic associations, as reflected by fewer TUNEL-positive cells and modulation of apoptosis-related proteins, including decreased Bax and cleaved caspase-3 and increased Bcl-2. NDRG4 overexpression was associated with reduced cytotoxic edema in the perihematomal region, which primarily arises from cell swelling following injury. Moreover, activation of the PI3K/Akt/GSK3β pathway by NDRG4 overexpression may further contribute to edema attenuation, given the pathway’s role in regulating cell survival, ionic balance, and endothelial barrier integrity in the CNS^44,45^. TUNEL staining and Western blot analyses further indicated that NDRG4 overexpression reduced apoptosis in the perihematomal region. It should be noted that the apoptotic rate in the sham + ad-NDRG4 group appeared slightly higher than in the sham group; however, this difference was not statistically significant (*p* = 0.8322; Fig. 5). This small variation is most likely attributable to the stereotaxic viral injection procedure itself rather than a pro-apoptotic effect of NDRG4. Baseline apoptosis occurs in normal brain tissue, and stereotaxic injection—through needle penetration and local viral delivery—can induce transient mechanical injury, local inflammation, and vector-associated tissue responses, which may modestly increase TUNEL-positive cells. Previous studies have reported that intracerebral administration of viral vectors can elicit focal neuroinflammation and apoptosis^46^, supporting the notion that the injection procedure alone can cause minor increases in apoptosis. This likely accounts for the slightly elevated apoptotic rate observed in the sham + ad-NDRG4 group in our study. Since apoptosis is a major form of cell death in this area after ICH^10,11^, these results provide mechanistic evidence that NDRG4 may contribute to cellular protection following hemorrhagic injury.

Our data also indicate that the PI3K/Akt/GSK3β pathway may be involved in mediating the protective effects of NDRG4. Akt is a serine/threonine kinase critical for cell survival and apoptosis regulation^44^. Its activation occurs through PI3K-dependent phosphorylation, which inhibits downstream pro-apoptotic mediators, including glycogen synthase kinase-3β (GSK3β)^47^. GSK3β, highly expressed in the central nervous system, participates in apoptosis in multiple cell types^48,49^. Its activity is regulated by site-specific phosphorylation: phosphorylation at Tyr216 enhances its activity, whereas phosphorylation at Ser9 suppresses it^50–52^. In our study, ICH reduced the phosphorylation of Akt(Ser473) and GSK3β (Ser9), consistent with suppression of the pathway. By contrast, NDRG4 overexpression was associated with restoration of Akt phosphorylation and increased inhibitory phosphorylation of GSK3β at Ser9, which may be associated with modulation of pro-apoptotic signaling. These observations are consistent with prior studies reporting that PI3K/Akt activation is linked to neuroprotection in ischemic stroke^45^, subarachnoid hemorrhage^53–57^, and ICH^43,52,58–61^.

The potential involvement of PI3K/Akt signaling in the observed associations with NDRG4 overexpression was further supported by experiments using Wortmannin, a selective PI3K inhibitor. In the presence of Wortmannin, the effects of NDRG4 overexpression on Akt and GSK3β phosphorylation, as well as on apoptosis markers, were reduced, indicating that the PI3K/Akt/GSK3β pathway may be involved in the observed associations. These findings not only reinforce the importance of this pathway in ICH injury, but also highlight a potential link between NDRG4 and PI3K/Akt activity that has not been previously described.

Taken together, our results provide new insight by connecting NDRG4 expression with apoptotic regulation and PI3K/Akt/GSK3β signaling after ICH. This expands earlier findings from ischemia and glioblastoma models and supports a broader role for NDRG4 in modulating cell-survival pathways. However, several limitations should be acknowledged. First, this study utilized only adult male rats, and potential sex- or age-related differences in NDRG4 function were not addressed, which may limit the generalizability of the findings. Second, although NDRG4 overexpression demonstrated protective effects, complementary loss-of-function approaches—such as knockdown or knockout models—were not performed. This omission limits our ability to determine whether NDRG4 is endogenously required for ICH pathophysiology or whether its effects are dose-dependent. Third, our experiments largely focused on the acute phase (24 h post-ICH), capturing maximal edema and apoptosis but not later stages of recovery; thus, the long-term impact of NDRG4 on functional outcomes remains unclear. Fourth, while our results support the involvement of the PI3K/Akt/GSK3β pathway in NDRG4-associated cellular protection, only wortmannin was used to inhibit PI3K. Additional approaches, such as siRNA-mediated knockdown or the use of direct Akt/GSK3β inhibitors, were not employed. Given the multifactorial nature of apoptotic mechanisms after ICH and the complex interplay among signaling pathways, the contribution of other cascades—including MAPK, NF-κB, and autophagy—cannot be excluded, suggesting that NDRG4-associated cellular protection may involve multiple interacting pathways. Fifth, behavioral assessments were performed only in Experiment 3 to evaluate the functional effects of NDRG4 overexpression. Experiments primarily designed to investigate molecular mechanisms (e.g., Experiment 4 involving wortmannin treatment) did not include behavioral testing. This limits our ability to directly correlate pathway modulation with neurological recovery. Future studies incorporating behavioral evaluations alongside molecular interventions are needed to better elucidate the functional relevance of these signaling mechanisms. Additionally, while our data suggest that improvements in motor function are largely driven by central neuroprotection, we cannot fully exclude contributions from spontaneous peripheral recovery, and the current study design does not allow precise separation of these effects. Sixth, we did not perform co-localization analyses using neuronal, astrocytic, or microglial markers to determine which cell populations were transduced by Ad-NDRG4. Consequently, the observed associations cannot be attributed specifically to neurons. The beneficial effects should therefore be interpreted as general perihematomal cellular protection rather than neuron-specific cellular protection. Finally, NDRG4 is not a specific PI3K agonist, and its neuroprotective actions are likely mediated through multiple mechanisms beyond PI3K activation.

In summary, our findings indicate that NDRG4 expression decreases after ICH, and that pre-injury overexpression of NDRG4 was associated with reduced perihematomal edema, histopathological injury, apoptosis, and early neurological deficits. These associations are potentially linked with modulation of the PI3K/Akt/GSK3β pathway. By linking NDRG4 to early cellular injury mechanisms after ICH, our study extends current understanding and highlights NDRG4 as a potential modulator of secondary brain injury.

## Conclusion

In summary, our study provides experimental evidence that NDRG4 overexpression was associated with reduced brain edema, lower apoptosis in the perihematomal region, and improved early neurological outcomes in a rat model of ICH. These associations appear to be linked, at least in part, with modulation of apoptotic signaling pathways, possibly involving activation of the PI3K/Akt/GSK3β axis. Several limitations should be noted. First, this work was conducted in an animal model, and its relevance to human ICH remains uncertain. Second, the mechanisms underlying NDRG4-mediated effects are likely multifactorial, whereas our analysis focused primarily on the PI3K/Akt/GSK3β pathway. Third, the study assessed only the early phase after ICH, and potential long-term effects warrant further investigation. Finally, because cell-type–specific localization was not examined, the observed effects should be interpreted as general perihematomal cellular associations rather than neuron-specific effects. Taken together, these findings suggest that NDRG4 may be involved in modulating secondary brain injury after ICH, but additional mechanistic and translational studies are required to clarify its functions and therapeutic potential.

## Supplementary Information

Below is the link to the electronic supplementary material.

Supplementary Material 1

Supplementary Material 2

Supplementary Material 3

Supplementary Material 4

Supplementary Material 5

Supplementary Material 6

Supplementary Material 7

## Data Availability

The datasets used and/or analyzed during the present study are available from the corresponding author on a reasonable request. Xiaoyan Wang and Liqiang Liu confirm the authenticity of all the raw data during this study.
