# Supplementary material for: NDRG4 overexpression is associated with reduced apoptosis after intracerebral hemorrhage via the PI3K/Akt/GSK3β signaling pathway
Source: Sci Rep. 2026 Jan 3;16:3374. doi: 10.1038/s41598-025-33247-5 (PMC12834981; doi:10.1038/s41598-025-33247-5)
Supplement: Supplementary file 3 — Supplementary Material 3 [file 41598_2025_33247_MOESM3_ESM.pdf]

Supplementary Materials 3

Full-length gel and blot images corresponding to Figure 6 are provided below.

A

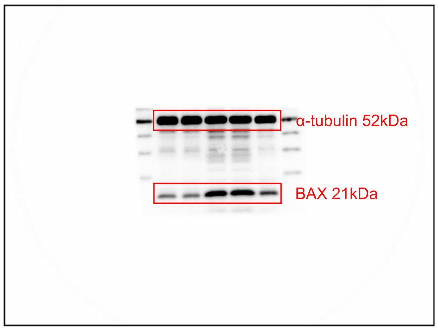

B

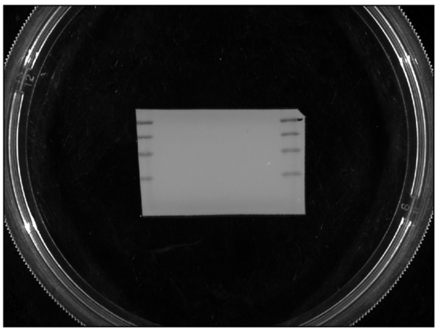

C

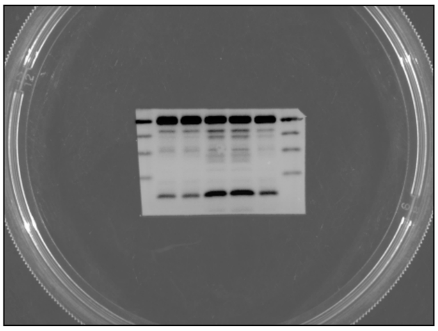

BAX and α-tubulin were obtained from the same gel under a single exposure.

(A) Full-length western blot;

(B) Full-length gel;

(C) Merged image of blot and gel.

A

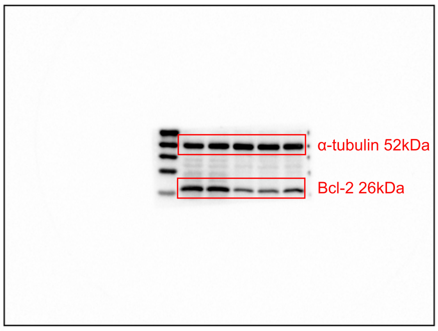

B

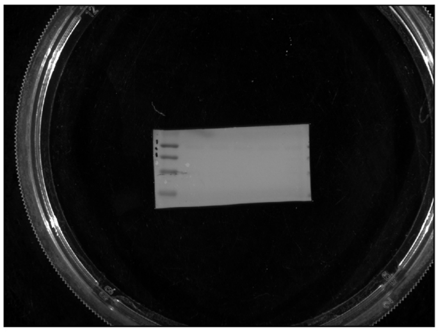

C

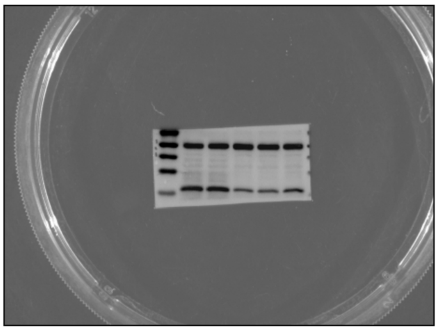

Bcl-2 and α-tubulin were obtained from the same gel under a single exposure.

(A) Full-length western blot;

(B) Full-length gel;

(C) Merged image of blot and gel.

A

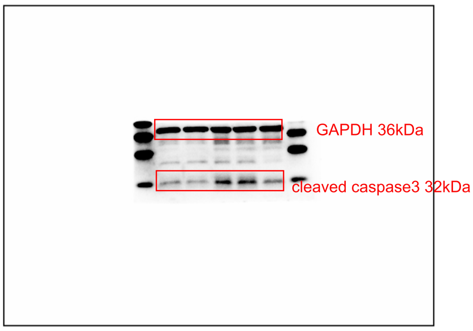

B

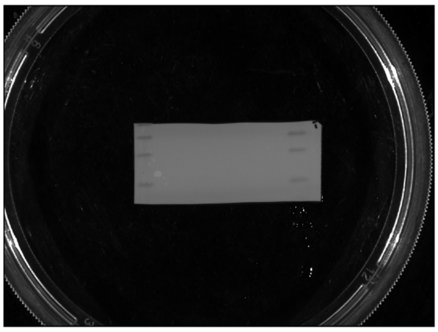

C

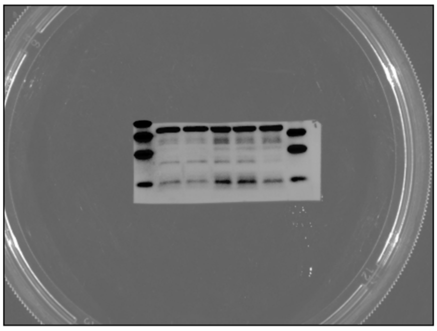

Cleaved Caspase3 and GAPDH were obtained from the same gel under a single exposure.

(A) Full-length western blot;

(B) Full-length gel;

(C) Merged image of blot and gel.
