# Supplementary material for: NDRG4 overexpression is associated with reduced apoptosis after intracerebral hemorrhage via the PI3K/Akt/GSK3β signaling pathway
Source: Sci Rep. 2026 Jan 3;16:3374. doi: 10.1038/s41598-025-33247-5 (PMC12834981; doi:10.1038/s41598-025-33247-5)

Supplementary Materials 5

Representative macroscopic brain sections following collagenase-induced intracerebral hemorrhage.

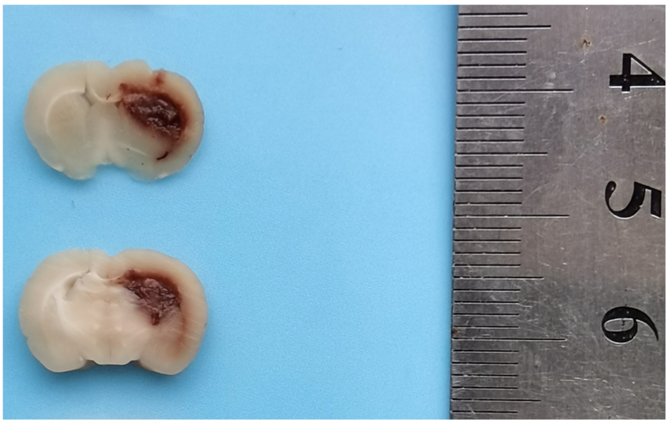

Supplement: Supplementary file 5 — Supplementary Material 5 [file 41598_2025_33247_MOESM5_ESM.pdf]
