# Supplementary material for: NDRG4 overexpression is associated with reduced apoptosis after intracerebral hemorrhage via the PI3K/Akt/GSK3β signaling pathway
Source: Sci Rep. 2026 Jan 3;16:3374. doi: 10.1038/s41598-025-33247-5 (PMC12834981; doi:10.1038/s41598-025-33247-5)
Supplement: Supplementary file 6 — Supplementary Material 6 [file 41598_2025_33247_MOESM6_ESM.pdf]

Table S1. Summary of experimental groups and mortality rate in the study.

Experimental Groups	Neurological test	WB	PCR	Tunel	Water content	Exclusion	Mortality (%)	Subtotal
**Experimental 1**								
Sham		6	6					6
ICH (12h,24h,48h,72h)		24	24			1	2(7.41%)	27
**Experimental 2**								
Sham		6						6
Sham+ad-NC		6						6
Sham+ad-NDRG4		6						6
ICH		6					1(14.28%)	7
ICH+ad-NC		6					1(14.28%)	7
ICH+ad-NDRG4		6				1	1(12.50%)	8
**Experimental 3**								
Sham	6	6		6	6			24
Sham+ad-NDRG4	6	6		6	6			24
ICH	6	6		6	6	1	4(13.79%)	29
ICH+ad-NC	6	6		6	6	2	3(10.34%)	29
ICH+ad-NDRG4	6	6		6	6	1	3(10.71%)	28
**Experimental 4**								
Sham		6						6
ICH		6					1(14.28%)	7
ICH+ad-NDRG4		6				1	1(14.28%)	7
ICH+ad-NDRG4+Vehicle		6				1	1(12.50%)	8
ICH+ad-NDRG4+Wortmannin		6					1(14.28%)	7
**Total**	30	96	30	30	30	8	18(7.44%)	242

ICH, intracerebral hemorrhage; WB, ad-NC, adenoviral negative control vector; ad-NDRG4, adenoviral vector encoding NDRG4
