# Supplementary material for: NDRG4 overexpression is associated with reduced apoptosis after intracerebral hemorrhage via the PI3K/Akt/GSK3β signaling pathway
Source: Sci Rep. 2026 Jan 3;16:3374. doi: 10.1038/s41598-025-33247-5 (PMC12834981; doi:10.1038/s41598-025-33247-5)
Supplement: Supplementary file 7 — Supplementary Material 7 [file 41598_2025_33247_MOESM7_ESM.pdf]

The ARRIVE guidelines 2.0: author checklist

ARRIVE Item	Description / Requirement	Reported in Manuscript
**1. Study Design**	For each experiment, provide brief details of study design including: (a) the groups being compared, including control groups; (b) the experimental unit (e.g., a single animal, litter, or cage of animals).	Four independent experiments:<br>**Exp 1:** Temporal NDRG4 expression, 5 groups (Sham, ICH-12h, ICH-24h, ICH-48h, ICH-72h, n=6/group).<br>**Exp 2:** NDRG4 overexpression validation, 6 groups (Sham, Sham+ad-NC, Sham+ad-NDRG4, ICH, ICH+ad-NC, ICH+ad-NDRG4, n=6/group).<br>**Exp 3:** Effects on ICH outcomes, 5 groups (Sham, Sham+ad-NDRG4, ICH, ICH+ad-NC, ICH+ad-NDRG4); subsets used for neurological, brain water content, TUNEL, WB analyses (6 rats per group per assay).<br>**Exp 4:** PI3K/Akt/GSK3β pathway, 5 groups (Sham, ICH, ICH+ad-NDRG4, ICH+ad-NDRG4+Vehicle, ICH+ad-NDRG4+Wortmannin, n=6/group).<br>Experimental unit = single rat.
**2. Sample Size**	(a) Exact number of experimental units allocated to each group, total number in each experiment, and total animals used; (b) how sample size was decided, including any a priori calculation.	Total 242 rats across 4 experiments. Each group contained 6 rats per assay; extra animals included to compensate for mortality/exclusions. No formal power calculation; sample size based on previous experience/published studies. Exact n per group reported in Table S1.
**3. Inclusion and Exclusion Criteria**	(a) Criteria for including/excluding animals or data points, specify if pre-defined; (b) report any exclusions per group; (c) exact n per analysis.	**Inclusion:** body weight 200–250 g, normal neurological function, successful ICH induction.<br>**Exclusion:** failed ICH induction, unexpected death unrelated to procedure, technical failures.<br>8 rats excluded for failed ICH; 18 rats died (7.44% mortality). Reported in Table S1 and Methods 2.1–2.2.
**4. Randomization**	State whether randomisation was used; method; strategy to minimise confounders (order, cage location, etc.).	Rats randomly assigned to groups. Order of surgery, behavioral testing, and tissue collection randomized; cage location/treatment order balanced when possible.
**5. Blinding**	Who was aware of group allocation at different stages (allocation, conduct, outcome assessment, analysis).	Behavioral assessments, histology (TUNEL), WB, RT-PCR performed by blinded investigators. Group allocation

		during surgery known only to surgeon performing procedure.
**6. Outcome Measures**	(a) Define all outcome measures; (b) specify primary outcome for hypothesis-testing studies.	**Primary outcomes:** neurological function (forelimb placement, Bederson, Longa), brain water content, apoptosis (TUNEL), protein/gene expression (WB, RT-PCR).<br>**Secondary outcomes:** PI3K/Akt/GSK3β signaling.
**7. Statistical Methods**	(a) Details of statistical methods including software; (b) methods to check assumptions and actions if not met.	GraphPad Prism 9. Student's t-test (2 groups), one-way ANOVA (multiple groups), repeated-measures mixed-model ANOVA for longitudinal data. Sphericity assessed (Mauchly's test), Greenhouse–Geisser correction if violated. Normality/homogeneity evaluated; Kruskal–Wallis tests when appropriate. $P<0.05$ considered significant.
**8. Experimental Animals**	(a) Species, strain, sex, age, weight; (b) further relevant info (health, immune status, genotype, prior procedures).	242 male Sprague Dawley rats, 10–12 weeks old, 200–250 g, healthy, pathogen-free, no prior procedures. Housed at 22 ±2 °C, 50–60% humidity, 12 h light/dark cycle, ad libitum food/water.
**9. Experimental Procedures**	(a) What, how, and what used; (b) when/how often; (c) where including acclimatisation; (d) why (rationale).	ICH via stereotactic collagenase IV injection (1 μL, 0.3 U/μL) into right striatum; sham = saline. Adenoviral vectors stereotaxically injected 3 days prior (0.1 mL, 1×10^8 U/mL). Analgesia. Perfusion/euthanasia, tissue collection, TUNEL, WB, RT-PCR, brain water content performed per published protocols. Procedures detailed to allow replication.
**10. Results**	(a) Summary/descriptive statistics per group with variability; (b) effect size with CI if applicable.	Results, Mortality and Exclusion; Table S1; mortality 18/242 (7.44%)
